# Supplementary material for: Hoxa5 Inhibits the Proliferation and Induces Adipogenic Differentiation of Subcutaneous Preadipocytes in Goats
Source: Animals (Basel). 2022 Jul 21;12(14):1859. doi: 10.3390/ani12141859 (PMC9311789; doi:10.3390/ani12141859)

Figure S1. Western Original Image

Full original blots used for Figure 2B. Each blot membrane was cut based on the standard band positions and then incubated with the appropriate antibodies.
The bands in the article are marked by red lines.

Figure 2B

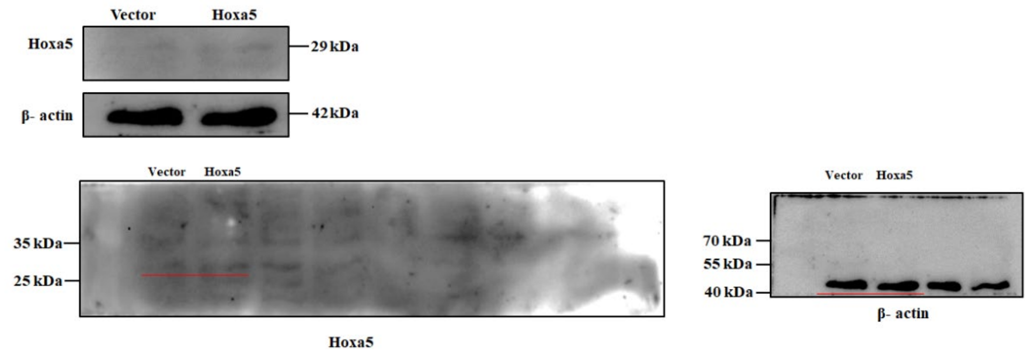

Full original blots used for Figure 3B. Each blot membrane was cut based on the standard band positions and then incubated with the appropriate antibodies.
The bands in the article are marked by red lines.

Figure 3B

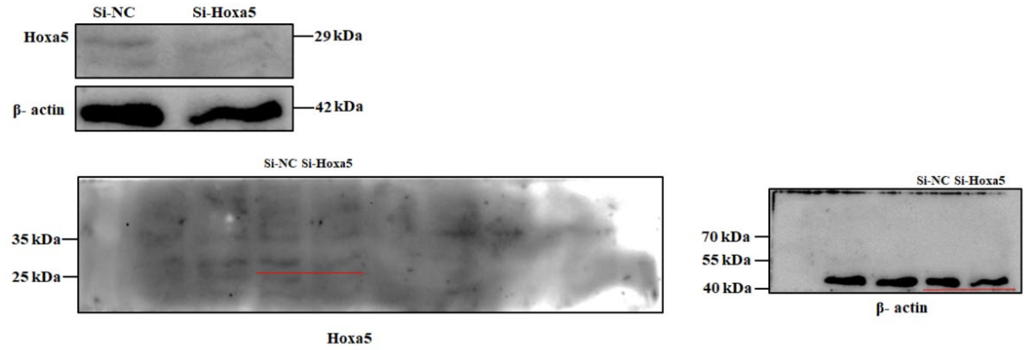

Supplement: Supplementary file 1 [file animals-12-01859-s001.zip › Western Original Image.pdf]
